# Supplementary material for: Resting state MEG oscillations show long-range temporal correlations of phase synchrony that break down during finger movement
Source: Front Physiol. 2015 Jun 17;6:183. doi: 10.3389/fphys.2015.00183 (PMC4469817; doi:10.3389/fphys.2015.00183)
Supplement: Supplementary file 1 [file DataSheet1.PDF]

Supplementary Material: Resting state MEG oscillations show long-range temporal correlations of phase synchrony that break down during finger movement

Maria Botcharova [1,2], Luc Berthouze [3,4], Matthew J. Brookes [5], Gareth R. Barnes [6] and Simon F. Farmer [2,*]

[1] *Centre for Mathematics and Physics in the Life Sciences and Experimental Biology, University College London, London, UK*
[2] *Institute of Neurology, University College London, London, UK*
[3] *Centre for Computational Neuroscience and Robotics, University of Sussex, Falmer, UK*
[4] *Institute of Child Health, University College London, London, UK*
[5] *Sir Peter Mansfield Magnetic Resonance Centre, School of Physics and Astronomy, University of Nottingham, Nottingham, UK*
[6] *The Wellcome Trust Centre for Neuroimaging, University College London, London, UK*

Correspondence*:
Simon F Farmer
Institute of Neurology, University College London, London, UK, s.farmer@ucl.ac.uk

1 COMPARISON OF DFA EXPONENT BETWEEN STITCHED AND NON-STITCHED TIME SERIES FOR LONG TIME SERIES (N=10^6)

In this section, we provide empirical evidence to support the assertion that, provided suitable window sizes are considered, DFA is insensitive to stitching. For a number of FARIMA[p,d,q] configurations – for memory, a FARIMA process makes it possible to control both the Hurst exponent through parameter d=H-0.5 and the amount of short-term/long-term correlations through parameters p and q. –, we compare the application of DFA to two time series: the first is generated by a single FARIMA[p,d,q] process of length N=10^6 samples; the second results from stitching n=10 FARIMA[p,d,q] processes of length $\lfloor N/n \rfloor$ samples. DFA calculations are as per the manuscript, i.e., the maximum window size is $\lfloor N/n \rfloor$ samples, i.e., the length of a single trial.

Figure 1 shows our results for a self-similar FARIMA process with Hurst exponent 0.7. For the sake of completeness, we also include data showing that stitching is also possible when non self-similar FARIMA configurations are considered. Figure 2 corresponds to a FARIMA process with some degree of short-term correlation – this typically leads to parabolic fluctuation plots. Figure 3 corresponds to a FARIMA process contaminated (through linear superposition) by a slow sinusoidal input – this typically leads to a piece-wise linear fluctuation plot. Note that we only provide those plots to show the qualitative similarity between plots. In either case, these plots would be discarded by ML-DFA as not being acceptable fluctuation plots.

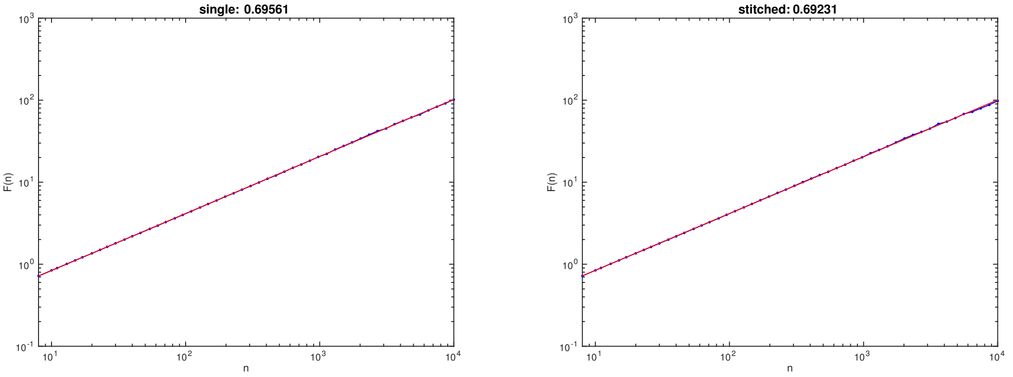

Figure 1. FARIMA(0,0.2,0) scenario. N=10^6 samples. Left: DFA fluctuation plot for single time series. Right: DFA fluctuation plot for stitched time series (n=10 records). Difference in exponent: 0.0033.

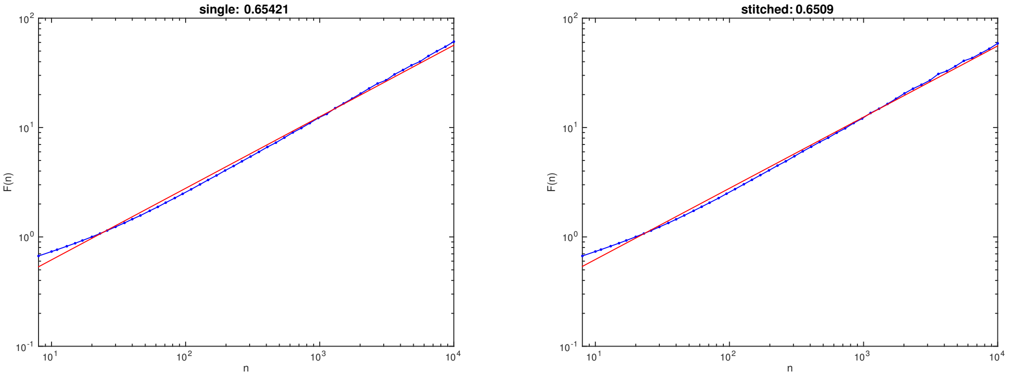

Figure 2. FARIMA(0,0.2,0.4) scenario. N=10^6 samples. Left: DFA fluctuation plot for single time series. Right: DFA fluctuation plot for stitched time series (n=10 records).

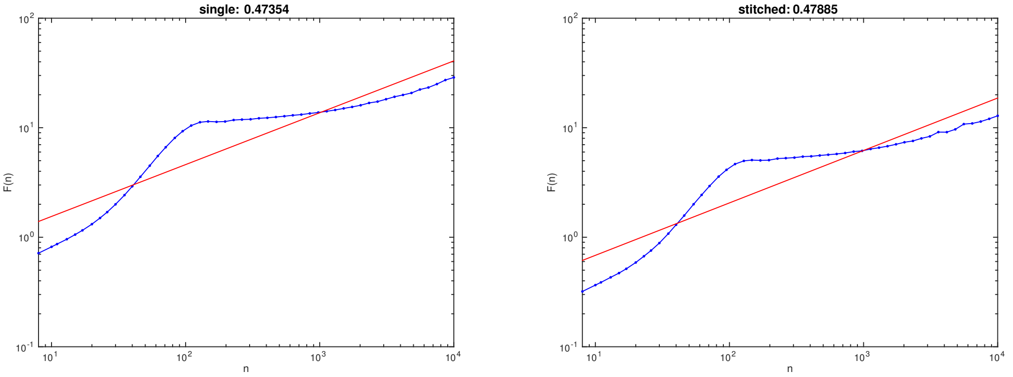

Figure 3. FARIMA(0,0.0,0.0) with slow sinusoidal noise scenario. N=10^6 samples. Left: DFA fluctuation plot for single time series. Right: DFA fluctuation plot for stitched time series (n=10 records).

2 COMPARISON OF DFA EXPONENT BETWEEN STITCHED AND NON-STITCHED TIME SERIES FOR SHORT TIME SERIES (N=180000)

As it may be argued that the above only holds because long time series are considered, in this section, we replicate the above scenarios with N set to the number of samples in the experimental time series used in the manuscript, namely: N=180000 (10 trials of 30s at sampling frequency Fs=600Hz).

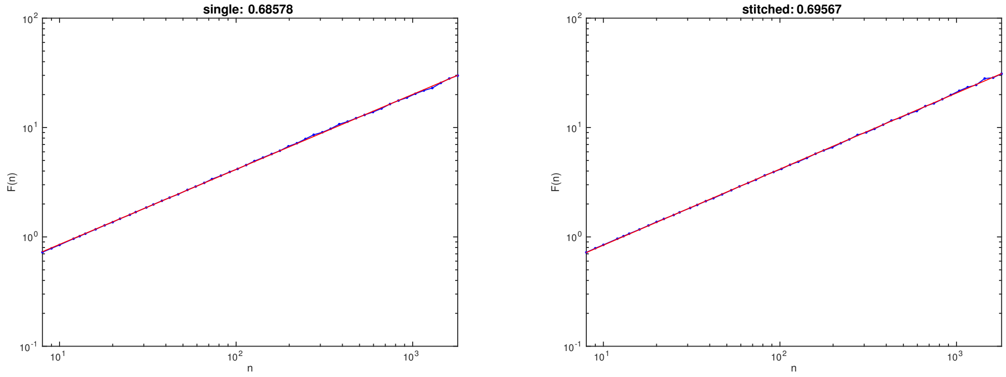

Figure 4. FARIMA(0,0.2,0) scenario. N=180000 samples. Left: DFA fluctuation plot for single time series. Right: DFA fluctuation plot for stitched time series (n=10 records). Difference in exponent: 0.0099.

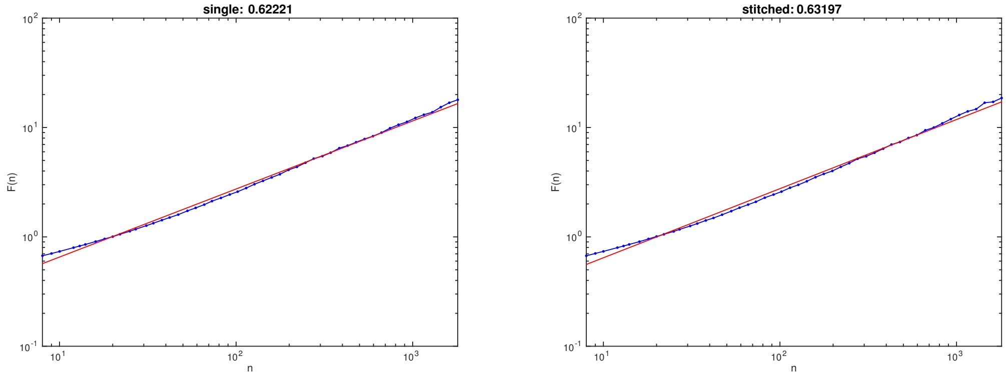

Figure 5. FARIMA(0,0.2,0.4) scenario. N=180000 samples. Left: DFA fluctuation plot for single time series. Right: DFA fluctuation plot for stitched time series (n=10 records).

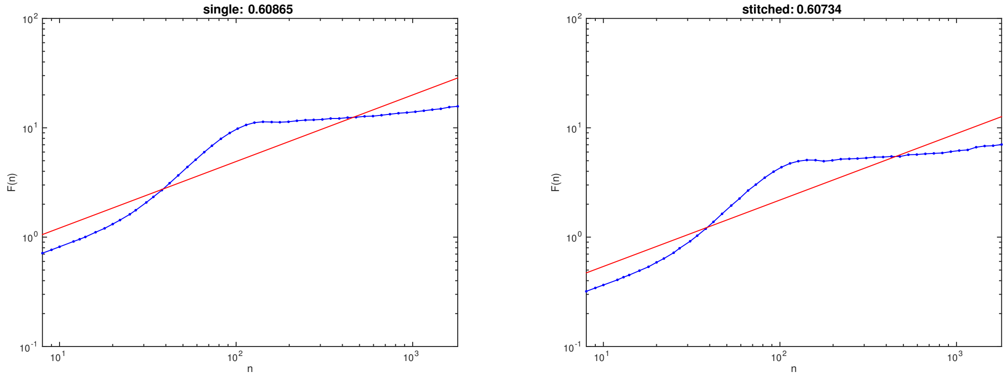

Figure 6. FARIMA(0,0.0,0.0) with slow sinusoidal noise scenario. N=180000 samples. Left: DFA fluctuation plot for single time series. Right: DFA fluctuation plot for stitched time series (n=10 records).
